# Crystal structure, biochemical and cellular activities demonstrate separate functions of MTH1 and MTH2

**DOI:** 10.1038/ncomms8871

**Published:** 2015-08-04

**Authors:** Megan Carter, Ann-Sofie Jemth, Anna Hagenkort, Brent D. G. Page, Robert Gustafsson, Julia J. Griese, Helge Gad, Nicholas C. K. Valerie, Matthieu Desroses, Johan Boström, Ulrika Warpman Berglund, Thomas Helleday, Pål Stenmark

**Affiliations:** 1 Department of Biochemistry and Biophysics, Stockholm University, S-106 91 Stockholm, Sweden; 2 Science for Life Laboratory, Division of Translational Medicine and Chemical Biology, Department of Medical Biochemistry and Biophysics, Karolinska Institutet, S-171 21 Stockholm, Sweden

## Abstract

Deregulated redox metabolism in cancer leads to oxidative damage to cellular components including deoxyribonucleoside triphosphates (dNTPs). Targeting dNTP pool sanitizing enzymes, such as MTH1, is a highly promising anticancer strategy. The MTH2 protein, known as NUDT15, is described as the second human homologue of bacterial MutT with 8-oxo-dGTPase activity. We present the first NUDT15 crystal structure and demonstrate that NUDT15 prefers other nucleotide substrates over 8-oxo-dGTP. Key structural features are identified that explain different substrate preferences for NUDT15 and MTH1. We find that depletion of NUDT15 has no effect on incorporation of 8-oxo-dGTP into DNA and does not impact cancer cell survival in cell lines tested. NUDT17 and NUDT18 were also profiled and found to have far less activity than MTH1 against oxidized nucleotides. We show that NUDT15 is not a biologically relevant 8-oxo-dGTPase, and that MTH1 is the most prominent sanitizer of the cellular dNTP pool known to date.

## Abstract (web summary)

Dysfunctional redox regulation in cancer can damage dNTPs so inhibiting dNTP pool sanitizing enzymes, such as MTH1, is a potential cancer treatment. Here, Carter *et al.* characterize MTH2 (NUDT15) and show that it is not a dNTP sanitizer, and so is unlikely to influence the efficacy of MTH1 inhibitors.

## Graphical abstract

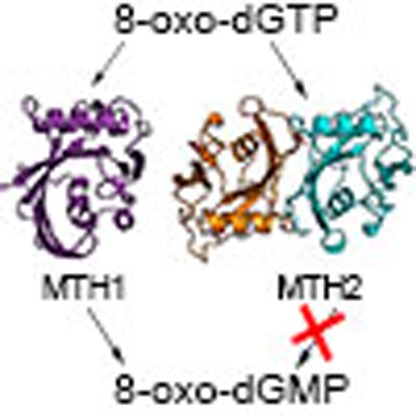

Despite advances in targeted therapies and personalized medicine, DNA damaging treatments such as radio- and chemotherapy remain the primary anticancer treatment modality^1^. These treatments induce damage indiscriminately and often result in dose-limiting side effects that reduce patients' quality of life^1^,^2^,^3^. Because of the unspecific nature of DNA damaging agents, advanced metastasized cancers are rarely cured and cancer remains one of the most common causes of death.

Altered metabolic and redox activities in cancer cells increase the production of reactive oxygen species that can cause DNA damage^4^,^5^. While bases in DNA are protected by the double helix and nucleosome packing, free deoxyribonucleoside triphosphates (dNTPs) are unprotected and therefore more easily damaged^6^. Thus, for cancer cells with deregulated reactive oxygen species, nucleotide pool sanitizing enzymes are critically important, as damaged dNTPs can be incorporated into the DNA thus inducing mutations and DNA damage.

First discovered in *Escherichia coli*, the NUDIX protein MutT was identified as a sanitizer of the oxidized dNTP pool^7^. MutT selectively hydrolyses 8-oxo-dGTP to 8-oxo-dGMP, thus preventing 8-oxo-guanine from being incorporated into DNA and subsequent A:T→C:G transversion mutations^7^. In this way, MutT serves as a ‘house-keeping' enzyme that maintains DNA integrity by preventing 8-oxo-dG-induced mutations. The NUDIX hydrolase superfamily is widespread, found in >250 species in eukaryotes, bacteria and archaea. Members of this family consist mainly of pyrophosphohydrolases that act upon nucleoside diphosphatates linked to other moieties, X (NUDIX)^8^. The human genome has 24 NUDIX hydrolase genes and at least 5 pseudogenes that display considerable substrate diversity. The substrate diversity of NUDIX family proteins is to be expected given that the identifying sequence motif encompasses the metal binding and catalytic site, whereas substrate recognition and selectivity are carried out by variable regions within the superfamily. Multiple functional human homologues of MutT have been suggested, including NUDT1 (MutT homologue 1, MTH1; ref. 9), NUDT15 (MutT homologue 2, MTH2; refs 10, 11) and NUDT18 (MutT homologue 3, MTH3; ref. 12). NUDT17 is a close familial relative of MTH1 whose activity has yet to be explored^12^.

The MTH1 protein is the best characterized of the suggested MutT homologues. We and others have recently demonstrated that MTH1 is critical for the survival of cancer cells where oxidative damage to dNTPs is prominent^13^,^14^,^15^. Interestingly, MTH1 is not essential in non-transformed cells^13^,^14^, and MTH1^−/−^ knockout mice are viable with a similar lifespan as wild-type mice^16^. For these reasons, MTH1 inhibition has shown great potential as a novel strategy to treat cancer—a concept coined ‘cancer phenotypic lethality'^13^. Despite the promise of first-in-class MTH1 inhibitors, there is a concern that NUDT15 or other dNTP sanitizing enzymes may compensate for lost MTH1 activity. Hence, we sought to investigate other enzymes that sanitize the dNTP pool and determine whether these enzymes might be targeted to induce cancer phenotypic lethality or overcome resistance to MTH1 inhibitors.

NUDT15 is reported to convert 8-oxo-dGTP and 8-oxo-dGDP to 8-oxo-dGMP^10^, whereas NUDT18 is known to hydrolyse 8-oxo-dGDP to 8-oxo-dGMP^12^. The activity of NUDT15 towards 8-oxo-dGTP has led to the assumption that it may contribute to sanitizing the oxidized dNTP pool. While NUDT15 loss has been shown to increase mutation frequency 1.5-fold upon exposure to 8-oxo-dGTP^10^,^11^, the *in vitro* activity of NUDT15 against 8-oxo-dGTP is much lower than the activity of MTH1 towards 8-oxo-dGTP or 2-OH-dATP^12^,^17^. Despite rather strong claims supporting NUDT15 as an 8-oxo-dGTP hydrolase, extensive profiling of human NUDT15 is noticeably lacking within the scientific literature.

Here, we solve the first crystal structure of NUDT15 and elucidate major structural differences compared with MTH1, especially in the enzymatic pocket. We demonstrate that NUDT15 has considerably lower activity to oxidized guanine species than to the undamaged nucleotides. Furthermore, knockdown of NUDT15 does not influence the level of 8-oxo-dG incorporation into DNA or cancer cell survival, suggesting that the enzyme does not sanitize 8-oxo-dGTP in cells. We also test NUDT17 and NUDT18 for their activity against a panel of oxidized and canonical NUDIX substrates and show that these enzymes have poor activity against oxidized dNTPs compared with MTH1. Altogether, we conclude that MTH1 is the major sanitizer of the oxidized dNTP pool with little or no overlapping functions of NUDT15, NUDT17 and NUDT18.

## Results

### MTH1 is a major sanitizer of 8-oxo-dGTP and 2-OH-dATP

**Figure 1:**
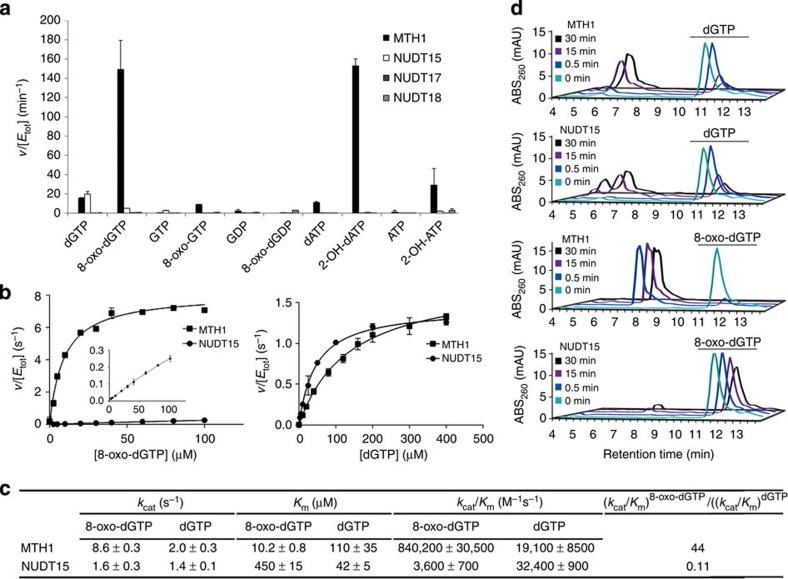
Comparison of NUDIX protein activity with nucleotide substrates. (**a**) Nucleotide substrate (50 μM) was incubated with 5–500 nM NUDIX protein depending on enzyme. Hydrolysis was monitored by detecting phosphate generated. The depicted data are representative of two independent experiments showing the same result. Data are presented as *v* (hydrolysed substrate (μM) per minute) per [enzyme] (μM). (**b**) Saturation curves and kinetic parameters of MTH1- and NUDT15-mediated hydrolysis of 8-oxo-dGTP (left) and dGTP (right). NUDT15 (8 nM) or MTH1 (0.25 nM) was incubated with 8-oxo-dGTP at concentrations ranging from 0 to 100 μM in assay buffer, and initial rates were determined in duplicate. Inset highlights NUDT15 activity on a smaller activity scale. NUDT15 (8 nM) and MTH1 (2 nM) were incubated with dGTP in assay buffer ranging from 0 to 400 μM, and initial rates were determined in duplicate. Data are presented as *v* (hydrolysed substrate (μM) per second) per [enzyme] (μM), and are representative of data collected from at least two independent experiments. (**c**) Kinetic parameters of MTH1 and NUDT15 for dGTP and 8-oxo-dGTP hydrolysis. The Michaelis–Menten equation was applied to saturation curves using the GraphPad Prism software and kinetic parameters were calculated. Data presented are average±s.d. from two independent experiments. (**d**) HPLC chromatograms showing the activity of NUDT15 and MTH1 against 8-oxo-dGTP and dGTP. The depicted data are representative of three independent experiments and show that MTH1 rapidly hydrolyses 8-oxo-dGTP; however, no significant activity is observed with NUDT15. Both MTH1 and NUDT15 can hydrolyse dGTP.

The MTH1 protein is a well-studied NUDIX protein that hydrolyses oxidized adenosine and guanosine triphosphates, thus preventing their incorporation into DNA. Similar to MTH1, other NUDIX proteins have proposed roles as dNTP pool sanitizers and may function in concert with MTH1 or may act as compensatory enzymes if the activity of MTH1 is lost. In the search for potential anticancer targets that sanitize the oxidized nucleotide pool, we investigated other MutT homologues with reported activity towards oxidized nucleotides (NUDT15 and NUDT18). NUDT17 was also analysed as it is closely related to the human MutT homologues^12^. These enzymes were purified and screened for activity against oxidized and canonical nucleoside triphosphates and diphosphates (substrate abbreviations found in Supplementary Table 1) using a malachite green-based assay (Fig. 1a and Supplementary Fig. 1). While MTH1 demonstrated strong activity towards 8-oxo-dGTP, 2-OH-ATP and 2-OH-dATP, none of the other proposed dNTP pool sanitizing enzymes showed appreciable activity with oxidized nucleotides.

Of these enzymes, NUDT15 showed the second highest activity against 8-oxo-dGTP (Fig. 1a), but had much stronger activity towards 2′-Deoxyguanosine-5′-triphosphate (dGTP) (Fig. 1a–c). To more thoroughly compare the activity of NUDT15 and MTH1, kinetic analysis of each enzyme using 8-oxo-dGTP and dGTP as substrates was conducted. Each protein was incubated with dGTP (0–400 μM) or 8-oxo-dGTP (0–100 μM) in assay buffer at pH 7.5, pyrophosphate formation was measured and initial rates were calculated (Fig. 1b). Kinetic parameters were determined using the Michaelis–Menten equation and are displayed in Fig. 1c. While MTH1 prefers 8-oxo-dGTP to dGTP (*k*_cat_/*K*_m_=840,200 and 19,100 M^−1^ s^−1^, respectively), NUDT15 had an opposite selectivity profile (*k*_cat_/*K*_m_=3,600 and 32,400 M^−1^ s^−1^ towards 8-oxo-dGTP and dGTP, respectively). MTH1 is a more efficient catalyst of 8-oxo-dGTP hydrolysis compared with NUDT15, which is reflected in a considerably higher *k*_cat_ value (36-fold higher), and an ∼44-fold lower *K*_m_ value for this substrate. MTH1 displays a slightly higher *k*_cat_ value compared with NUDT15 towards dGTP, but NUDT15 has a lower *K*_m_ value resulting in a similar catalytic efficiency of dGTP hydrolysis for the two enzymes. High-performance liquid chromatography (HPLC) experiments confirmed that NUDT15 had minimal activity against 8-oxo-dGTP, whereas MTH1 and NUDT15 had nearly equal activity against dGTP (Fig. 1d). Thus, our data show that NUDT15 has a much lower activity towards 8-oxo-dGTP than MTH1 and prefers dGTP over 8-oxo-dGTP. This was not the case for NUDT17 and NUDT18, which show higher activity with oxidized nucleotides compared with the corresponding canonical nucleotides (Supplementary Fig. 1b). This activity, however, is very low. Taken together, these data suggest that while other NUDIX enzymes show some biochemical activity against oxidized nucleoside diphosphates and triphosphates, the activity of these enzymes are very modest in comparison with the activity of MTH1 towards 8-oxo-dGTP and 2-OH-dATP.

### The structure of NUDT15 is similar but distinct from MTH1

**Figure 2:**
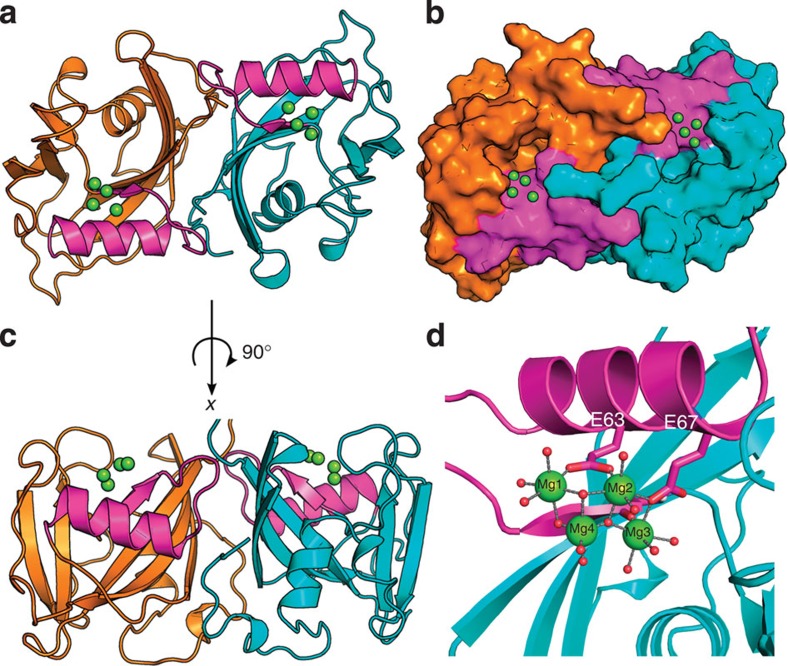
Crystallographic structure of dimeric NUDT15. (**a**) Top view of Nudt15 dimer (chain A in cyan and chain B in orange) ribbon representation with Mg coordination, and the NUDIX box highlighted in magenta. (**b**) Surface representation of NUDT15 dimer looking into the putative binding pocket. (**c**) Side view of NUDT15 dimer. (**d**) Four Mg ions in coordination (green spheres) with multiple waters (red spheres), E63, E67 and carbonyl oxygen of G47 residues in the NUDIX box (magenta).

Although NUDT15 is similar in sequence to MTH1, we have demonstrated that NUDT15 has a remarkably low activity towards 8-oxo-dGTP. To gain insight into the substrate preference of NUDT15, we determined the structure of the human NUDT15 protein (Fig. 2). NUDT15 was crystallized in sitting drop vapour diffusion experiments at 18 °C after 2 days in the conditions described in the Methods section. Crystals were analysed using the beamline 14.1 at Bessy, Germany, at 100 K. The structure was solved in a P1 space group at a resolution of 1.8 Å. Structure and refinement statistics can be found in Table 1.

The most striking difference between MTH1 and NUDT15 is that NUDT15 forms a homodimer (Fig. 2a–c). Analysis of the NUDT15 dimer interface with PISA (protein interfaces, surfaces and assemblies, an EBI service)^18^ demonstrates a solvation-free energy gain of 16.2 kcal mol^−1^ and a *P* value upon formation of the dimer of 0.186, with an interface area of 1,578.5 Å^2^. Size-exclusion chromatography and small-angle X-ray scattering (SAXS) experiments confirm that the dimeric assembly observed in the crystal structure is also present in solution (Supplementary Fig. 2).

The overall structure of the NUDT15 monomer follows a typical NUDIX fold with an α-helix, β-sheet, α-helix arrangement, where the helices reside on opposing sides of a mixed β-sheet (Fig. 2a,c). NUDT15 also has multiple small regions of 3_10_-helices. The NUDIX box (GX_5_EX_7_REUXEEXGU) is located in the first α-helix relative to the N terminus and contains residues responsible for magnesium coordination and substrate hydrolysis. Four magnesium ions are present in the NUDIX box region (Fig. 2d and Supplementary Fig. 3). Three out of four magnesium ions exhibited octahedral coordination: Mg1 coordinates Glu63 and 5 water molecules; Mg2 coordinates Glu63, Glu67 and 4 water molecules; and Mg3 coordinates Glu67, the carbonyl oxygen of Glu47 and 4 water molecules. The fourth magnesium ion, Mg4, has trigonal bipyramidal coordination (equatorial coordination angles that average 119.6°) and coordinates five water molecules, two of which are bridged with Mg1 and one is bridged with Mg2. While the presence of four magnesium ions may not be physiologically relevant, the structural differences between MTH1 and NUDT15 may have profound impact on the biological roles of these enzymes.

### NUDT15 substrate pocket poorly accommodates 8-oxo-dGTP

**Figure 3:**
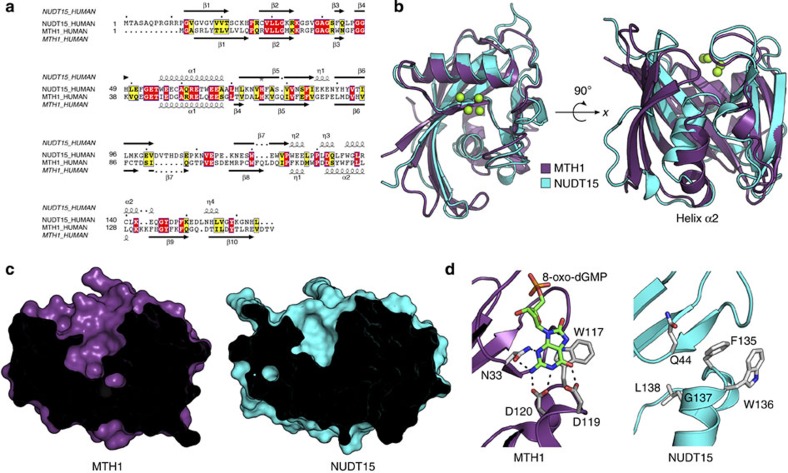
Structural comparison and sequence alignment of NUDT15 and MTH1. (**a**) Sequence alignment of human NUDT15 with human MTH1 (29% overall sequence identity). The secondary structure of NUDT15 is displayed above the sequence alignment and that of MTH1 below the alignment. Sequence similarity is represented by yellow boxes, strict sequence identity in red boxes, beta sheets (β) as arrows, alpha-helices (α) and 3_10_-helices (η) as squiggles. (**b**) Superimposition of MTH1 (purple) and NUDT15 (chain A, cyan) with the NUDT15 Mg^2+^ coordination is shown. Major structural deviation is observed in helix α2 that affects the depth of the putative binding pocket. (**c**) Comparison of NUDT15 and MTH1 binding pocket depth. Cut view of surface representation highlights the variation in binding pocket depth of MTH1 (purple) and Nudt15 (cyan). (**d**) Comparison of NUDT15 and MTH1 binding pocket composition. MTH1 helix α2 residues W117, P118, D119, D120 and N33 (grey) are involved in 8-oxo-dG (green) binding. NUDT15 helix α2 residues F135, W136, G137, L138 and Q44 (grey) form the bottom of the putative binding pocket and are unable to make the equivalent hydrogen bonding network observed in MTH1 binding to 8-oxo-dGMP.

While NUDT15 and MTH1 share considerable sequence homology (Fig. 3a) and are similar in overall structure (Fig. 3b), there are pronounced differences within the substrate binding sites that impact substrate recognition. Structural superimposition of NUDT15 and MTH1 reveals a change in the orientation of the second α-helix relative to the N terminus (helix α2) that forms the base of the binding pocket. This divergence leads to a 5.7 Å shift at the C-terminal end of the helix^19^, resulting in a considerably shallower putative binding pocket for NUDT15 (Fig. 3c). The compositions of the individual binding pockets are also markedly different. In MTH1, Asp119 and Asp120 make up the base of the binding pocket and form hydrogen bonds with 8-oxo-dGMP (Fig. 3d). In NUDT15, Trp136 and Gly137 occupy equivalent positions but are unable to form the same hydrogen bonding network. Residue Asn33 in MTH1, which is important for substrate recognition^19^, is partially conserved with Gln44 in the NUDT15 structure. However, Gln44 of NUDT15 is not in the same orientation and instead forms hydrogen bonds with residues of the adjacent β7 and β2 beta sheets. MTH1 residue Trp117 is involved in pi-stacking interactions with the substrate and is functionally conserved by residue Phe135 in NUDT15. The active site differences are highlighted in Fig. 3d, illustrating that NUDT15 and MTH1 have distinct binding site features that confer distinct substrate preferences.

### NUDT15 and MTH1 have distinct biological roles

**Figure 4:**
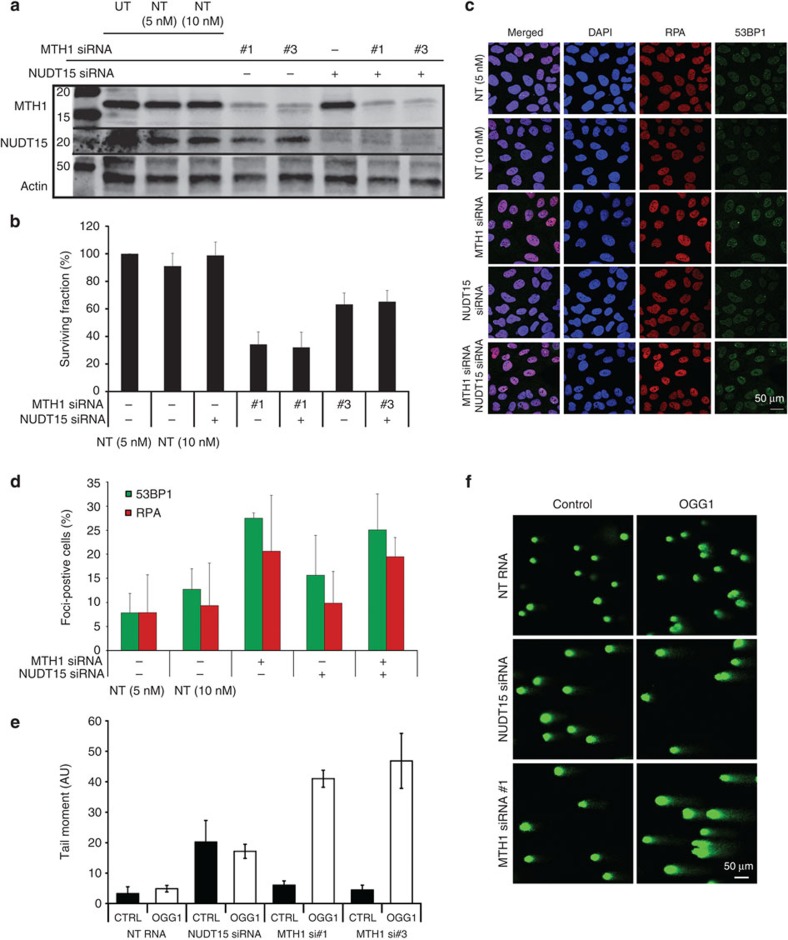
Effect of NUDT15 knockdown on clonogenic survival, DNA damage responses and 8-oxo-dG levels in DNA. (**a**) Western blot showing knockdown of MTH1 or NUDT15 in U2OS cells 96 h after transfection. (**b**) U2OS cells depleted of MTH1, NUDT15 or both by siRNA were seeded for clonogenic survival. Colonies were stained after 10 days with methylene blue and colonies were counted by eye; (*n*=2). (**c**) Representative immunostainings. After NUDT15, MTH1 or combination of both RPA foci, 53BP1 foci and DAPI (4,6-diamidino-2-phenylindole) were visualized using immunofluorescence. (**d**) Quantification of 53BP1- and RPA-positive cells (>5 foci per cell) from **c**; (*n*=2). (**e**) Quantification of the tail moment after MTH1 and NUDT15 knockdown; (*n*=2). (**f**) Representative pictures of comets formed after MTH1 or NUDT15 depletion by siRNA. Lysed cells were treated with OGG1 or buffer control. UT, untransfected; NT, non-targeting siRNA control. Data shown as average±s.d.

Since NUDT15 had only limited activity against 8-oxo-dGTP *in vitro*, we analysed whether removal of NUDT15 from cells would increase the 8-oxo-dG content of DNA, induce any DNA damage markers or impact cancer cell survival. Interestingly, NUDT15 depletion using short interfering RNA (siRNA) had no effect on the survival of several cancer cell lines (U2OS, NTUB1/p, A375, H460, MCF7 and SW480; Fig. 4a,b and Supplementary Fig. 4a,b). This is in strong contrast to MTH1, whose depletion results in significantly impaired cancer cell survival (Fig. 4a,b). Furthermore, NUDT15 siRNA depletion did not influence the survival of MTH1 siRNA-depleted cells, indicating no additive or synergistic effect of combined knockdown (Fig. 4b). In addition, no alterations in cell cycle distribution could be detected after NUDT15 knockdown (Supplementary Fig. 4c,d), and the NUDT15 expression showed no fluctuations throughout the cell cycle (Supplementary Fig. 4e). It is established that MTH1 siRNA depletion results in an increase in DNA damage markers^13^. Here we confirm that MTH1 siRNA depletion induces RPA and 53BP1 foci formation, but find no such effect after NUDT15 depletion (Fig. 4c,d). Altogether, these data suggest that NUDT15 is not required for prevention of 8-oxo-dGTP incorporation into DNA. To test this directly, we determined the presence of 8-oxo-dG in DNA using the modified comet assay after depleting NUDT15 by siRNA and compared the effect with MTH1 knockdown. In line with our findings that NUDT15 has a poor 8-oxo-dGTPase activity, no increase of 8-oxo-dG was observed in DNA following depletion of NUDT15 by siRNA as was observed when depleting MTH1 (Fig. 4e,f). Even though siRNA depletion does not lead to complete removal of the protein, these data further corroborate that NUDT15 has a limited role in sanitizing the dNTP pool from 8-oxo-dGTP in cells, and that the cellular function is distinct from MTH1.

### Distinct activity profiles of MTH1 and NUDT15

**Figure 5:**
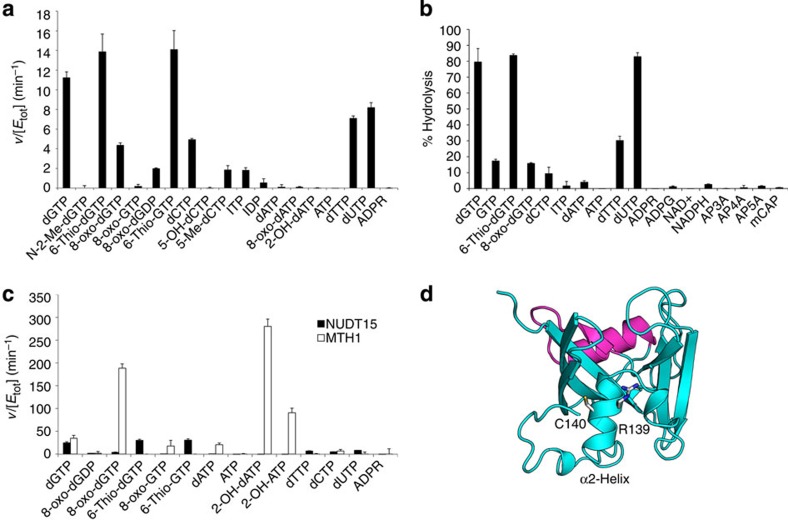
Activity of NUDT15 towards potential NUDIX family substrates. (**a**) Substrate hydrolysis after 30 min incubation at 22 °C was monitored by coupling the reaction to an excess of bovine alkaline phosphatase or *E. coli* pyrophosphatase and measuring inorganic phosphate using malachite green reagent. Data are presented as *v* (hydrolysed substrate (μM) per minute) per [NUDT15] (μM). Depicted data are mean±s.d. of triplicates. Experiment was performed twice with similar results. (**b**) Relative activity of NUDT15 by HPLC analysis. Activity of 10 nM NUDT15 protein against a panel of potential NUDIX substrates was measured by HPLC after 30 min incubation at 37 °C. Enzymatic activity was stopped by addition of trifluoroacetic acid (5%) and samples were ran under conditions indicated in Methods section. Per cent hydrolysis was calculated by subtracting the peak area at 30 min from the area at time 0 and dividing this by the area at time 0; (*n*=2). (**c**) Substrate hydrolysis after 20 min incubation at 22 °C with NUDT15 (black bars) or MTH1 (white bars). Substrate hydrolysis was monitored by coupling the reaction to phosphatase or pyrophosphatase and measuring the presence of inorganic phosphate as described above. Experiments were performed in duplicate and data are presented as *v* (hydrolysed substrate (μM) per minute) per [enzyme] (μM). The experiment was repeated with similar results. (**d**) Mutation of NUDT15 (cyan) Arg139 to Cys is implicated in thiopurine-induced leukopaenia. Arg139 is located at the base of the helix α2 adjacent to Cys140. The NUDIX box is shown in magenta.

As NUDT15 does not have a significant role in cellular 8-oxo-dGTP hydrolysis, we tested the activity of NUDT15 against a panel of potential substrates *in vitro*. Substrate hydrolysis was assessed using a malachite green assay (Fig. 5a) and HPLC (Fig. 5b). Strong correlation was observed between the two analysis techniques. To our knowledge, there have not been any reports of post-translational modifications of these enzymes, and we have not found any indications of such. However, the assays were carried out using recombinant, purified enzymes, and differences from the *in vivo* activity cannot be ruled out.

Importantly, when comparing the activity of NUDT15 to MTH1, it is clear that NUDT15 has a distinct selectivity profile from MTH1 (Fig. 5c). Unlike MTH1, NUDT15 was not active towards adenosine nucleotides. Of the substrates tested, low activity was observed towards dCTP, dTTP and 8-oxo-dGTP. The strongest activity was observed against dGTP, 6-thio-dGTP, 6-thio-GTP and dUTP. Two independent studies have recently identified NUDT15 as a pharmacogenetic determinant of thiopurine sensitivity^20^,^21^. The particular mutation of NUDT15 leading to sensitivity to thiopurine therapy (Arg139Cys) is located in helix α2, which forms the base of the substrate binding pocket (Fig. 5d). This mutation could lead to the formation of a disulfide bond with the adjacent cysteine residue (Cys140) that may impact the structure of the enzyme active site. Such structural perturbation would likely reduce NUDT15 hydrolysis of 6-thio-dGTP in the cell, causing increased sensitivity to thiopurine treatment.

## Discussion

Since the MTH1 protein is a validated target for anticancer treatments^13^,^22^, it is critically important to explore other proposed dNTP pool sanitation enzymes and assess their relevance as possible cancer therapeutic targets. Also, these enzymes may greatly influence the efficiency of MTH1 inhibitors in the clinic. Here we profiled enzymes suggested to sanitize the oxidized dNTP pool. Although our data confirm that NUDT15 and NUDT18 have the ability to hydrolyse 8-oxo-dGTP or 8-oxo-dGDP in biochemical assays, this activity was extremely weak when compared with MTH1 and is not likely to have physiological relevance. Of the proposed MutT homologues, NUDT15 had the second highest activity towards 8-oxo-dGTP, but displayed much higher activity towards canonical dGTP. In fact, NUDT15 displays a nearly 10-fold preference for dGTP rather than 8-oxo-dGTP as a substrate (*k*_cat_/*K*_m_=32,400 M^−1^ s^−1^ for dGTP and 3,600 M^−1^ s^−1^ for 8-oxo-dGTP). This is in stark contrast to MTH1 where the *k*_cat_/*K*_m_ is over 40 times greater for 8-oxo-dGTP than for dGTP. This may be especially relevant in a biological setting where the amount of 8-oxo-dGTP is expected to be only a small fraction of the total dGTP pool. The kinetic values obtained in our experiments are in agreement with those previously published^17^,^19^ for MTH1 activity. Our experiments were conducted at the physiological pH of 7.5 in which we detected a reaction rate of 8-oxo-dGTP hydrolysis by MTH1 (8.6 s^−1^) in between the published *k*_cat_ values at pH 7.2 (2.1 s^−1^) and pH 8.0 (12.3 s^−1^) while the *K*_m_ value is similar to that previously reported^17^. At pH 8, Fujikawa *et al.,* observe a 13.5-fold preference of 8-oxdGTP over dGTP as substrate for MTH1. This preference for 8-oxo-dGTP seems to be more pronounced at pH 7.5 based on the results presented here. No kinetic parameters of human NUDT15 for any substrate have been reported previously. However, Takagi *et al*.^12^ estimate that MTH1 is ∼40-fold more active with 8-oxo-dGTP than NUDT15, which is in agreement with our results.

Furthermore, while MTH1 depletion led to marked reduction in clonogenic survival and increased levels of 8-oxo-dG in DNA, siRNA knockdown of NUDT15 had no effect on cell cycle progression, clonogenic survival, 8-oxo-dG content of DNA or DNA damage. Altogether, these data call into question the importance of NUDT15 in 8-oxo-dGTP sanitation and suggest a non-vital role of NUDT15 in this realm.

The poor activity of NUDT15 towards 8-oxo-dGTP can be explained by studying the crystal structure. While NUDT15 and MTH1 share a similar overall fold, a shift in NUDT15 helix α2 results in distortion of the substrate binding pocket compared with MTH1. In addition, MTH1 and NUDT15 have different residues that make up the base of their active sites, thereby conferring different substrate recognition. MTH1 has no direct contacts with the 8-oxo group of 8-oxo-dGTP; however, the crystal structure and mutational studies have demonstrated the importance of Asp119 and Trp117 in 8-oxo-dGTP recognition^19^,^23^. It has been suggested that the 8-oxo modification results in electronic changes that strengthen the hydrogen bonding interactions and/or the base-stacking interactions of 8-oxo-dGTP compared with dGTP^19^,^24^. NUDT15 lacks similar hydrogen bonding capabilities and does not maintain the same stacking interaction provided by Trp117 of MTH1, so it does not exhibit the same affinity for the oxidized nucleotides.

Since it appears that NUDT15 is not primarily involved in 8-oxo-guanine metabolism, we analysed its activity against a panel of potential NUDIX substrates. We conducted two independent substrate screens: one monitoring the formation of pyrophosphate using a malachite green-based assay and the other monitoring substrate hydrolysis by HPLC. Both assays showed that NUDT15 had low activity towards dTTP, dCTP and 8-oxo-dGTP, and higher activity towards dGTP, 6-thio-dGTP and 6-thio-GTP. While these represent novel NUDT15 substrates, the activity of NUDT15 against these substrates was quite low in comparison with MTH1 activity against 8-oxo-dGTP and 2-OH-dATP. The activity of NUDT15 towards 6-thioguanine species is especially interesting given that a NUDT15 mutation was recently identified as a sensitivity factor in thiopurine therapy^20^,^21^. While our data support a role for NUDT15 in thiopurine metabolism, further investigation into this topic is of great interest, as thiopurines are used in the treatment of cancer and autoimmune disease.

In conclusion, we demonstrate that MTH1 is the major, and perhaps only, NUDIX family member with a cellular role as a sanitizer of the oxidized dNTP pool. We demonstrate that NUDT15 is structurally distinct from MTH1, explaining the different substrate profile of NUDT15. We show that NUDT15 has a nonessential role in the survival of cancer cells but may be a mediator of thiopurine therapeutic effectiveness.

## Methods

### Expression and purification of recombinant NUDIX proteins

The bacterial expression construct pNIC28hNUDT15 was a kind gift from the Structural Genome Consortium (Stockholm, Sweden). cDNAs encoding NUDT17 and NUDT18 were optimized for expression in *E. coli*, purchased from GeneART (Life Technologies) and subcloned into pET28a(+) (Novagen). The constructs were verified by sequencing. His-tagged MTH1, NUDT15, NUDT17 and NUDT18 were expressed in *E. coli* BL21 (DE3) T1R pRARE2 cells at 18 °C overnight after induction with 0.5 mM isopropyl-β-D-thiogalactoside. Cultures were grown in a LEX system, harvested and lysed using Immobilized metal ion affinity chromatography (IMAC) lysis buffer (100 mM HEPES (pH 8.0), 500 mM NaCl, 10 mM imidazole, 10% glycerol and 0.5 mM Tris(2-carboxyethyl)phosphine hydrochloride (TCEP)), and pulsed sonication was performed. His-tagged proteins were affinity purified using HisTrap HP (GE Healthcare) followed by gel filtration using HiLoad 16/60 Superdex 75 (GE Healthcare) in gel filtration buffer (20 mM HEPES (pH 7.5), 300 mM NaCl, 10% glycerol and 0.5 mM TCEP). The His-tag was removed by TEV protease or thrombin digestion and separated using a Ni-NTA column or gel filtration using HiLoad 16/60 Superdex 75 (GE Healthcare). Purity of protein preparations was examined on SDS–polyacrylamide gel electrophoresis gel followed by Coomassie staining and concentration was determined using NanoDrop. The correct mass of the protein preparations was confirmed using mass spectrometry analysis.

### Crystallization and structure determination

Full-length NUDT15 (20 mg ml^−1^) was crystallized in the presence of α-chymotrypsin (0.2 mg ml^−1^) in 20 mM HEPES (pH 7.5), 300 mM NaCl, 10% glycerol and 1 mM TCEP. Sitting drop vapour diffusion experiments at 18 °C were performed, and NUDT15 was mixed with reservoir solution (30% PEG3350, 0.1 M Tris (pH 8.5) and 0.05 M MgCl_2_) in a 1:1 or 1:2 ratio. Diffraction quality crystals appeared after 2 days, were extracted quickly without additional cryoprotectant and flash frozen in liquid nitrogen. Data collection was performed at beamline 14.1 at Bessy, Germany, at 100 K and wavelength 0.9 Å. Data reduction and processing were carried out using XDS (X-ray Detector Software)^25^ and programs from the CCP4 (ref. 26) suite. The structure was solved by molecular replacement of the template structure file with PDB ID 4KYX using MolRep^27^, and Arp/wARP^28^ was used for building the initial model, followed by iterative building cycles using the Refine program in Phenix^29^. The structure was solved in P1 space group owing to issues arising from the presence of pseudo-translation symmetry. Relevant statistics can be found in Table 1. Ramachandran outliers totalled 0.3% of the final structure, however, all of these were glycine residues. The coordinates and structure factors for the structure presented in this paper was deposited in the PDB under code 5BON.

### SAXS analysis

To prepare samples for SAXS measurements, NUDT15 was additionally purified via gel filtration on a Superdex 200 column (GE Healthcare), and concentrated to yield samples in a concentration range from 1.5 to 13.5 mg ml^−1^ in 20 mM HEPES-Na (pH 7.5), 300 mM NaCl, 10% (v/v) glycerol and 2 mM TCEP. The flowthrough of the concentration step was used as buffer reference for SAXS measurements. Samples were centrifuged immediately before measurement. SAXS data were collected at beamline I911-SAXS/Max II at an X-ray wavelength of 0.91 Å over an *s*-range of 0.01–0.5 Å^−1^. The momentum transfer *s* is defined as *s*=4*π* sin*θ*/*λ*, where 2*θ* is the scattering angle and *λ* is the X-ray wavelength. Scattering profiles of lysozyme, bovine serum albumin and alcohol dehydrogenase (Sigma-Aldrich) were measured as reference for molecular mass determination. The ATSAS package^30^ was used to process and analyse data. The radius of gyration (*R*_g_) was derived by the Guinier approximation [*I*(*s*)=*I*(0) exp(−*s*^2^*R*_g_^2^/3) for *s R*_g_<1.3]. The molecular masses of the solutes were determined by extrapolating the scattering intensities to zero angle and using a standard curve obtained from *I*(0) values and known molecular masses of the reference proteins. Independent estimates of the molecular mass were obtained from the hydrated volume of the particles. Theoretical scattering profiles of atomic resolution models were calculated and fitted to measured profiles with CRYSOL^31^. *Ab initio* models were reconstructed from the experimental data using the programs DAMMIF^32^ and GASBOR^33^, initially without imposing any symmetry or other restrictions on possible models. Since all models were clearly twofold symmetric, further models were calculated imposing twofold symmetry. Sixteen envelopes that were independently reconstructed with DAMMIF were aligned and averaged with SUPCOMB^34^ and DAMAVER^35^. An envelope representation was calculated using the Situs package^36^, which was also used to dock the atomic resolution model into the envelope.

### Size-exclusion chromatography

A 10/300 GL S75 column was equilibrated on Äkta Prime with 20 mM HEPES (pH 7.5), 300 mM NaCl, 10% glycerol and 0.5 mM TCEP. Loaded protein samples were diluted to 500 μl each within the same buffer and 1–2 mM TCEP. Three separate samples were run over the column: 0.48 mg MTH1, 0.24 mg NUDT15 and a combined sample of 0.24 mg MTH1 and 0.24 mg NUDT15.

### Enzyme activity assays

Hydrolase activity of MTH1, NUDT15, NUDT17 and NUDT18 was tested with 50 μM canonical and oxidized nucleotides in assay buffer (0.1 M Tris-acetate (pH 8.0), 40 mM NaCl, 10 mM MgAc, 1 mM dithiothreitol and 0.005% Tween-20) after incubation at 22 °C for 30 min. Enzyme concentrations used were as follows: MTH1, 5 nM; NUDT15, 25 nM; NUDT17, 500 nM; and NUDT18, 25 nM. Hydrolysis was monitored by coupling the reaction to an excess of *E. coli* pyrophosphatase (Sigma-Aldrich), 0.2 U ml^−1^, and measuring the absorbance at 630 nm after addition of malachite green reagent^37^ and incubation for 15 min with agitation. A phosphate standard curve was used to convert the absorbance to [Pi] (A_630_=0.01743 × [Pi] (μM)). Specific activity of NUDT15 was determined towards a panel of nucleotide substrates using 75 nM NUDT15 and 50 μM of each respective nucleotide after 30 min incubation at 22 °C in assay buffer. For direct comparison, NUDT15- and MTH1-specific activities were determined in parallel using 75 nM NUDT15 for all nucleotides except dGTP (where 15 nM was used) and 2 nM MTH1. Enzymes were incubated with 50 μM of respective nucleotides for 20 min using the same conditions as described above. Hydrolase activity was assayed by coupling the reaction to an excess of bovine alkaline phosphatase (Sigma-Aldrich), 2.5 U ml^−1^, for 8-oxo-dGDP, inosine diphosphate (IDP) and adenosine diphosphate ribose (ADPR) or *E. coli* pyrophosphatase, 0.2 U ml^−1^, (Sigma-Aldrich; the rest of the tested substrates), producing inorganic phosphate, followed by addition of the malachite green reagent and measuring the absorbance at 630 nm. To determine kinetic parameters, a kinetic analysis of NUDT15 and MTH1 activity using dGTP and 8-oxo-dGTP as substrates was performed. NUDT15 (8 nM) or MTH1 (2 nM) was incubated with dGTP in the assay buffer described above ranging from 0 to 400 μM for 10, 20 and 30 min in duplicates. NUDT15 (8 nM) or MTH1 (0.25 nM) was incubated with 8-oxo-dGTP ranging from 0 to 100 μM 8-oxo-dGTP for 10, 20 and 30 min in duplicates. Formed pyrophosphate or inoorganic diphospate (PPi) was detected using PPi Light inorganic pyrophosphatase assay from Lonza. A PPi standard curve ranging from 0 to 5 μM PPi was included on each assay plate and used to calculate produced PPi. Initial rates were calculated using linear regression and for the determination of kinetic parameters, the Michaelis–Menten equation was fitted to the initial rate data by nonlinear regression using Prism (GraphPad, La Jolla, CA). The enzyme-catalysed hydrolysis of nucleotide substrates was also monitored using an HPLC assay. HPLC conditions were adapted from Pollak *et al.*^38^. Briefly, HPLC eluents were prepared as follows. Buffer A: H_2_O, 10 mM K_2_HPO_4_, 2 mM tetrabutyl ammonium bromide, pH 7.0; and buffer B: 50% H_2_O, 50% HPLC grade acetonitrile, 10 mM K_2_HPO_4_, 2 mM tetrabutyl ammonium bromide, pH 5.5. Samples were run on a 5-μm C18 column, 4 × 125 mm with a flow rate of 1 ml min^−1^. Linear gradients from 0 to 50% B over 10 min followed by from 50 to 70% B over 10 min were used. The column was then washed with 100% B for 4 min and re-equilibrated to 100% A for 4 min. Absorbance detection at 260 nm was used to quantify the amount of substrates at the time points indicated. Substrate consumption was measured by HPLC after 30 min incubation at 37 °C in assay buffer (0.1 M Tris-acetate (pH 8.0), 40 mM NaCl, 10 mM MgAc, 1 mM dithiothreitol and 0.005% Tween-20) with 10 nM NUDT15 protein. Enzymatic activity was quenched by adding 10 μl of 5% solution of trifluoroacetic acid to 30 μl of the reaction mixture. All samples were then diluted with HPLC buffer A (40 μl), which facilitated loading onto the HPLC. Per cent hydrolysis was calculated by subtracting the peak areas at time 30 min from the area at time 0 and then dividing this by the area at time 0.

### Cell culture

U2OS, A375, HCT116, HEK293T, HeLa, H460, MCF7 and SW480 cells were maintained in a humidified incubator at 5% CO_2_ and 37 °C. Cell lines were cultured in the following media supplemented with 10% fetal bovine serum and penicillin/streptomycin: DMEM: MCF7, HEK293T, HeLa; DMEM glutamax: SW480, A375; RPMI: and H460; McCoy's: U2OS, HCT116. The cell lines were regularly checked for mycoplasma with MycoAlert Mycoplasma Detection Kit (Lonza). U2OS, HEK293T, HCT116, H460, MCF7 and HeLa were obtained from American Type Culture Collection, SW480 from Walter Bodmer (Oxford University) and A375 from Jonas Nilsson (University of Gothenburg and Sahlgrenska University Hospital). None of the used cell lines were listed on ICLAC or were known to be cross-contaminated.

### siRNA transfection

Cells were seeded to reach 50% confluence at the day of transfection. After 24 h, 10 nM siRNA was transfected using INTERFERin (Polyplus Transfection), as described by the manufacturer using a final concentration of 10 nM siRNA. As negative control, the ALLStars negative control from Qiagen was used. The following oligonucleotide sense-sequences were used: MTH1 siRNA no. 1 5′-GACGACAGCUACUGGUUUC-3′, MTH1 siRNA no. 3 5′-CGACGACAGCUACUGGUUU-3′ and for NUDT15 a pooled mix of 50% 5′-GGAUGUGACUCAUGAUUCATT-3′ and 50% 5′-CCUCAGUUGUGAAUUCUUUTT-3′. Target sequences of siRNAs used in Supplementary Fig. 4 are as follows:

Qiagen 4: 5′-CAGCAGTACTCTTCTCACTAA-3′

Qiagen 6: 5′-CTCAAGAGCCTTTCAAGGGTA-3′

Qiagen 7: 5′-CUGGUGUGAUAUUGUAAUATT-3′

Qiagen 8: 5′-CCAUCUAAUAUCUGUAUCATT-3′

Ambion 1: 5′-GGAUGUGACUCAUGAUUCATT-3′

Ambion 2: 5′-GAAGGAGAAUUACCAUUAUTT-3′

Ambion 3: 5′-CCUCAGUUGUGAAUUCUUUTT-3′

### Western blotting

Briefly, protein lysates in lithium dodecyl sulfate (LDS) loading buffer and Precision Plus Protein Dual Color Standards were separated on NuPage 4–15% gradient gels with MOPS running buffer (Life Technologies), transferred to nitrocellulose membranes and blocked with 5% bovine serum albumin (BSA) in tris-buffered saline and tween. Membranes were incubated with primary antibodies diluted in 5% BSA/TBS-T with agitation at 4 °C overnight followed by incubation with secondary antibodies diluted in 5% BSA/TBS-T for 1 h at room temperature. The antibody against actin (dilution factor 1:10,000; ab6276; mouse monoclonal) was obtained from Abcam. NUDT15 (dilution factor 1:500; SC84533; rabbit), cyclin B1 (dilution factor 1:1,000; SC594; rabbit) and cyclin E (dilution factor 1:1,000; SC198; rabbit) antibodies were bought from Santa Cruz Biotechnology, and MTH1 (dilution factor 1:1,000; NB100-109; rabbit polyclonal) from Novus Biologicals. Secondary antibodies against rabbit and mouse were obtained from LI-COR Biosciences. Protein bands were visualized with an Odyssey Fc Imager and analysed with Image Studio Software (LI-COR Biosciences). The uncropped images of western blots are provided in Supplementary Fig. 5.

### Clonogenic survival

Cells were transfected by siRNA as described above, and after 2 days 500 cells were trypsinized, counted and re-seeded into 10-cm Petri dishes. After 10 days, colonies were stained with 4% methylene blue in MeOH and counted positive if cell number exceeded 50 cells.

### Immunofluorescence

Cells were seeded on glass coverslips in 12-well formats 24 h after siRNA transfection. Ninety-six hours after transfection, cells were fixed with 3% paraformaldehyde in PBS and permeabilized with 0.1% Triton X-100 in PBS for 15 min. Before antibody staining, cells were blocked with 3% BSA in PBS for 45 min. Antibodies used were as follows: RPA (dilution factor 1:500; Cell Signalling; 2,208; rat) and 53BP1 (dilution factor 1:500; Abcam; ab36823; rabbit polyclonal). Alexa-conjugated secondary antibodies were diluted 1:500 and incubated for 2 h. DNA was counterstained with 4,6-diamidino-2-phenylindole. Quantification was done by manual counting of positive cells in the microscope (*n*>400 cells per sample). Images were taken with a Zeiss LSM 780 with a × 63 oil objective. For 5-ethynyl-2 deoxyuridine (EdU) analysis, U2OS cells were seeded into 96-well plates. After 96 h of transfection, 10 μg ml^−1^ EdU was added for 20 min as indicated by the manufacturer's instructions (Invitrogen). Plates were fixed and stained as indicated above. Images were acquired using the Operetta (PerkinElmer) and analysed using the Harmony software.

### Modified comet assay

After 5 days of siRNA transfection, U2OS cells were harvested by trypsinization, washed with 1 × PBS and resuspended to 1 × 10^6^ cells per ml in 1 × PBS. Cell suspension was diluted 1:5 in 1.2% low-melting agarose (37 °C). A volume of 80 μl of cell suspension was spread on agarose-coated fully frosted slides (Thermo Fisher Scientific) with the help of a coverslip. Cells were lysed overnight at 4 °C using a buffer containing 10 mM Tris (pH 10), 1.5 M NaCl, 0.1 M EDTA, 10% dimethylsulfoxide and 1% Triton X-100. After washing the slides three times with enzyme buffer (40 mM HEPES (pH 8.0), 0.1 M KCl, 0.5 mM EDTA and 0.2 mg ml^−1^ BSA), DNA was exposed to buffer alone or OGG1 (2.0 μg ml^−1^) for 45 min at 37 °C. Slides were washed in enzyme buffer, followed by incubation in alkaline electrophoresis buffer (40 mM HEPES (pH 8.0), 0.1 M KCl, 0.5 mM EDTA and 0.2 mg ml^−1^ BSA) for 30 min. The gel electrophoresis condition used was 300 mA, 25 V for 30 min in electrophoresis buffer. After neutralization (0.4 M Tris-HCl, pH 7.5), DNA was stained using SybrGold dye (Invitrogen) and images were taken with a × 10 objective of a Zeiss LSM 780 confocal microscope. Comets were analysed using CometScore software and tail moment was calculated as per cent DNA in the tail multiplied by the tail length.

### Propidium iodine staining

Seventy-two hours after siRNA treatment, cells were lysed and DNA was stained with a buffer containing 50 μg ml^−1^ propidium iodine, 20 mM Tris (pH 8.0), 100 mM NaCl, 0.1% NP40 and 20 μg ml^−1^ RNase. After 1 h incubation in the dark, DNA content was analysed on a Navios flow cytometer from Beckman Coulter.

### Thymidine block

U2OS cells were blocked with a double thymidine block. Thymidine (2 mM; Sigma) was added for 18 h followed by a 9 h period with media. For the second block, 2 mM thymidine was added for 17 h. Cells were released by washing with PBS and addition of media. Western blot samples were taken at the indicated time points.

## Additional information

**Accession codes:** The structure and structure factors of NUDT15 have been deposited in the Protein Data Bank with the ID 5BON.

**How to cite this article:** Carter, M. *et al.* Crystal structure, biochemical and cellular activities demonstrate separate functions of MTH1 and MTH2. *Nat. Commun.* 6:7871 doi: 10.1038/ncomms8871 (2015).

## Supplementary Material

Supplementary Information Supplementary Figures 1-5 and Supplementary Table 1

## Figures and Tables

**Table 1:** Data collection and refinement statistics.

	**PDB ID: 5BON**
*Data collection*	
Space group	P1
Cell dimensions	
*a*, *b*, *c* (Å)	60.8, 71.7, 82.8
*α*, *β*, *γ* (°)	90.1, 90.0, 73.08
Resolution (Å)	47.5–1.8 (1.9-1.8)
*R*_sym_ or *R*_merge_	4.9 (74.7)
*I/*σ*I*	12.95 (1.4)
Completeness (%)	95.8 (93.9)
Redundancy	1.8 (1.7)
	
*Refinement*	
Resolution (Å)	47.5–1.8
No. of reflections	118,828
*R*_work_/*R*_free_	19.2/22.0
No. of atoms	
Protein	10,053
Ligand/ion	31
Water	1,013
*B*-factors	
Protein	36.7
Ligand/ion	33.0
Water	40.8
R.m.s.d.	
Bond lengths (Å)	0.004
Bond angles (°)	0.93

R.m.s.d., root mean squared deviation.

Values in parentheses are for highest-resolution shell. A single crystal was used for data collection and structure refinement.
