# Supplementary material for: Crystal structure, biochemical and cellular activities demonstrate separate functions of MTH1 and MTH2
Source: Nat Commun. 2015 Aug 4;6:7871. doi: 10.1038/ncomms8871 (PMC4532830; doi:10.1038/ncomms8871)
Supplement: Supplementary Information — Supplementary Figures 1-5 and Supplementary Table 1 [file ncomms8871-s1.pdf]

Supplementary Information

Crystal structure, biochemical and cellular activities demonstrate separate functions of MTH1 and MTH2

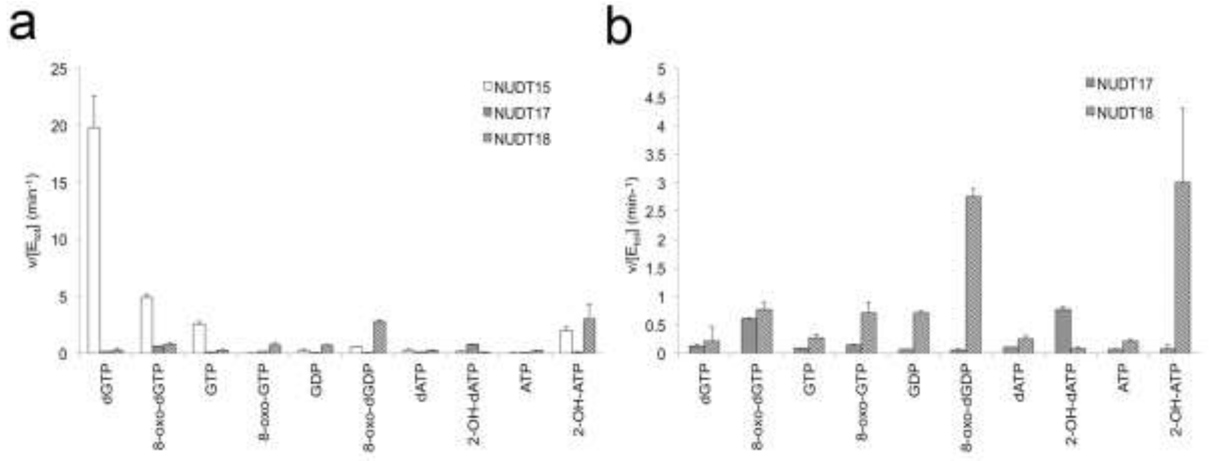

Supplementary Figure 1 Comparison of activities of NUDIX proteins with nucleotide substrates. Data shown are the same as shown in Figure 1a but without MTH1 and adjusted scale on the y-axis to more clearly visualize differences in activity of the NUDIX enzymes. (**a**) Data shows that NUDT15 has higher activity towards dGTP than towards 8-oxo-dGTP. (**b**) The same data as in (a) but without NUDT15 showing that NUDT18 has a preference for oxidized nucleotides although the activity is very low. NUDT17 displays low activity with 8-oxodGTP and 2-OH-dATP. Data presented are average ± standard deviation from two independent experiments.

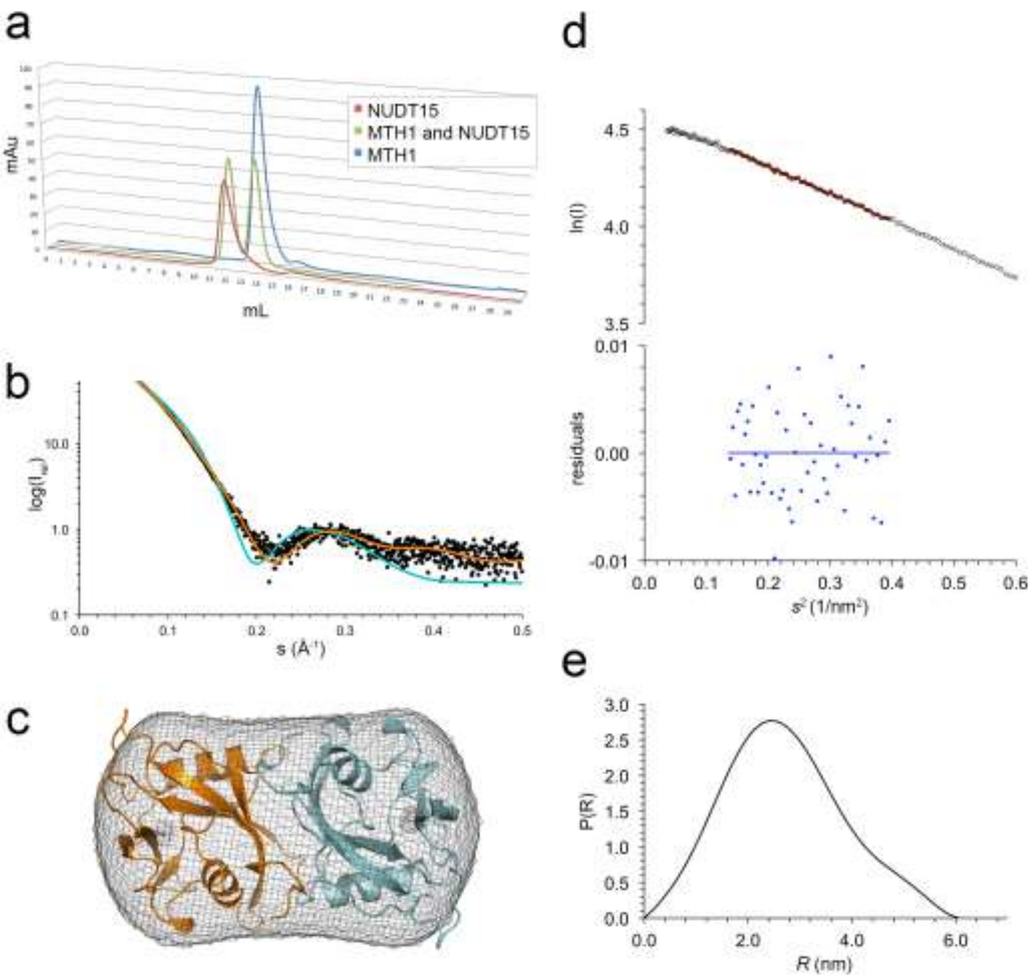

Supplementary Figure 2 NUDT15 is a dimer in solution. (**a**) Size exclusion chromatography elution profile of NUDT15 (red, elution peak at 11.96 ml), MTH1 (blue elution peak at 13.58 ml), and combined sample of NUDT15 and MTH1 (green, elution peaks at 11.96 and 13.62 ml). The elution profiles suggest that MTH1 (primary sequence-18 kDa) is a monomer in solution, as previously demonstrated, and NUDT15 (primary sequence-19 kDa) is a dimer. (**b**) Scattering profile of NUDT15 in solution (black) in comparison with the profile calculated from the crystallographic model of the monomer (cyan, Chi = 5.151) and dimer (orange, Chi = 1.617). The molecular mass of NUDT15 is estimated at 30 kDa which corresponds roughly to the molecular mass of a dimer. (**c**) Solution envelope reconstruction (gray mesh) of NUDT15, superimposed with the crystallographic model of the NUDT15 dimer (colored by chain). (**d**) Guinier plot (ln I(s) vs. s^2) of SAXS data of NUDT15 at 13.5 mg/ml with linear regression (for points shown in red) used for determination of R_g (R_g = 2.05 ± 0.09 nm). The lower plot (blue) shows the residuals of the linear regression. The sample shows no signs of aggregation or inter-particle repulsion. All samples in the concentration series yielded the same radius of gyration. (**e**) Distance distribution function calculated for SAXS data of NUDT15 at 13.5 mg/ml with Dmax = 6.25 nm, used for solution envelope reconstructions.

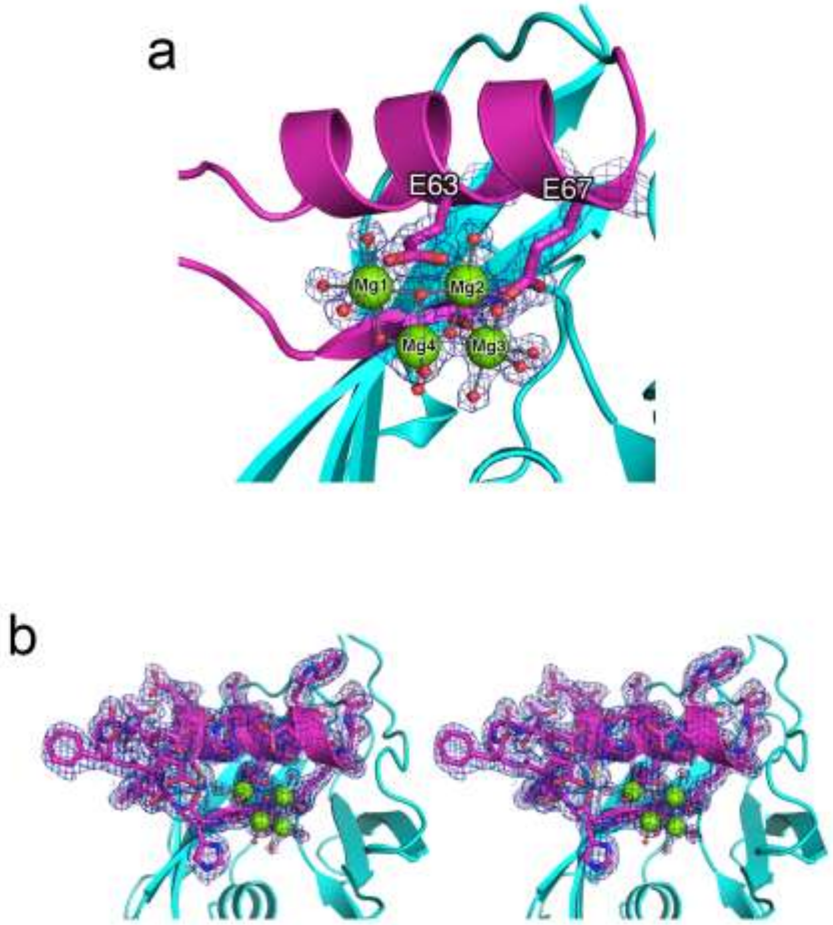

Supplementary Figure 3 Electron density of Mg coordination geometry observed in chain A of NUDT15. (**a**) Four Mg ions in coordination (green spheres) with multiple waters (red spheres), E63, E67, and carbonyl oxygen of G47 residues in the NUDIX box (magenta). Electron density 2Fo-Fc of Mg coordination is shown in blue mesh. Maps are contoured at 2.3 sigma. (**b**) Stereo image of electron density (2Fo-Fc) for the NUDIX box residues (magenta). Maps are contoured at 1.0 sigma.

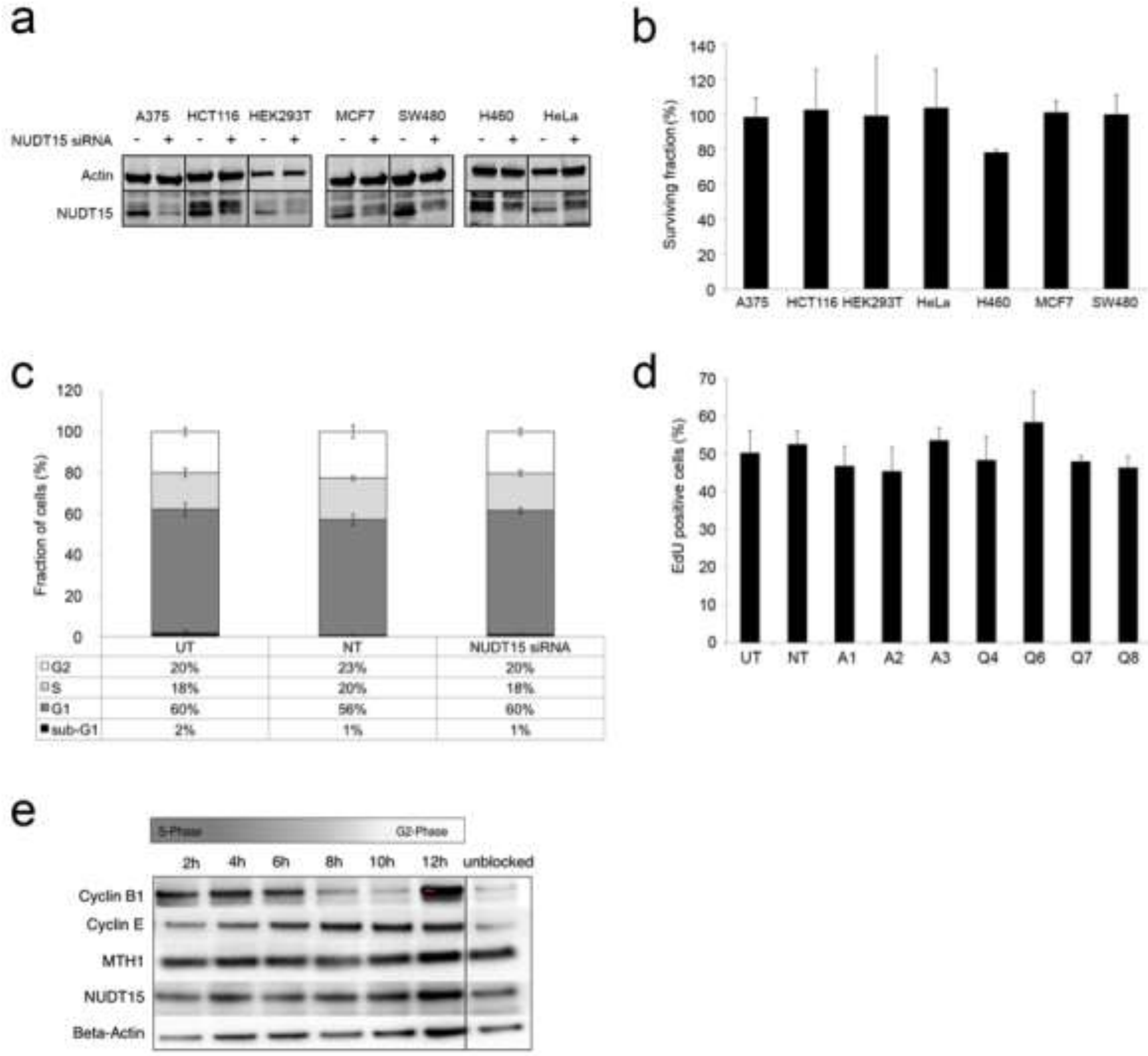

Supplementary Figure 4 NUDT15 is constantly expressed within the cell cycle and knockdown does not lead to cell cycle alterations nor cell death. (**a**) Western blot to confirm knockdown levels of NUDT15 in different cancer cell lines 96 hours after transfection. (**b**) Clonogenic survival after NUDT15 depletion in different cancer cell lines. (**c**) Cell cycle distribution by analyzing propidium iodine staining with FACS analysis 72h after NUDT15 knockdown. n=3. (**d**) EdU incorporation analysis 72h after knockdown of NUDT15 using 7 different siRNA constructs. n=2. (**e**) Expression of NUDT15 within the cell cycle. U2OS cells were blocked in G1 phase with a double thymidine block. Western blot samples were taken at the indicated time points after release. UT = untransfected sample, NT = non-targeting siRNA control. Error bars represent the average ± S.E.M.

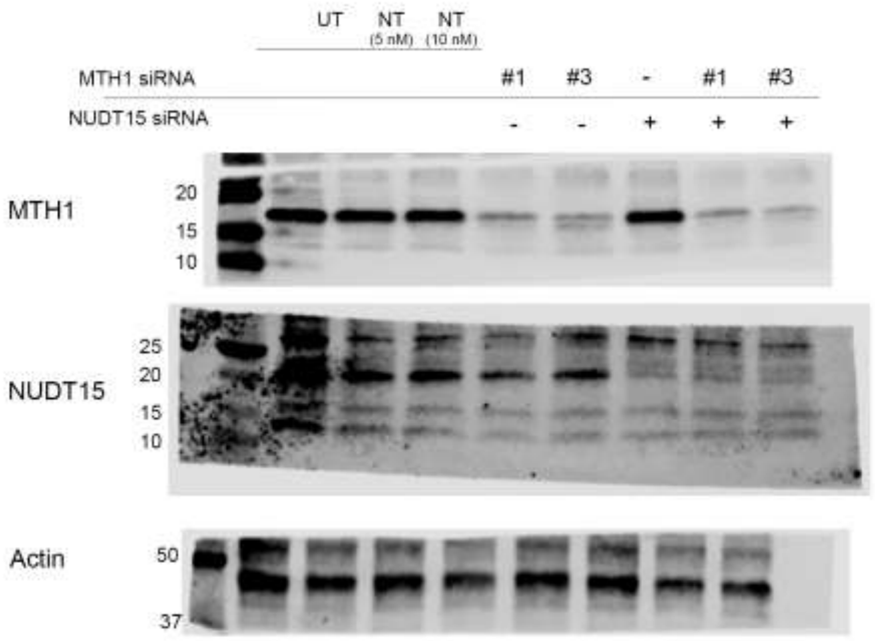

Supplementary Figure 5 Uncropped images of the western blot membranes shown in Figure 4b. UT= untransfected, NT= non-targeting siRNA. siRNA sequences used are described in the materials and methods section. The membranes were physically cut before blotting the individual pieces with various antibodies and imaged together. We are showing the individual pieces of relevant blots.

Supplementary Table 1: List of Abbreviations

2-OH-dATP	2-hydroxy-2’-deoxyadenosine-5’-triphosphate
5-Me-dCTP	5-methyl-2’-deoxycytidine-5’-triphosphate
5-OH-dCTP	5-hydroxy-2’-deoxycytidine-5’-triphosphate
6-thio-dGTP	6-thio-2’-deoxyguanosine-5’-triphosphate
6-thio-GTP	6-thioguanosine-5’-triphosphate
8-oxo-dATP	8-oxo-2’-deoxyadenosine-5’-triphosphate
8-oxo-dGTP	8-oxo-2´-deoxyguanosine-5´-triphosphate
8-oxo-GTP	8-oxo-guanosine-5'-triphosphate
AP3A	P1-(5´-Adenosyl) P3-(5´-adenosyl) triphosphate
AP4A	P1-(5´-Adenosyl) P4-(5´-adenosyl) tetraphosphate
AP5A	P1-(5´-Adenosyl) P5-(5´-adenosyl) pentaphosphate
ADPG	Adenosine-5´-diphosphoglucose
ADPR	Adenosine-5´-diphosphoribose
ATP	Adenosine-5´-triphosphate
dATP	2´-Deoxyadenosine-5´-triphosphate
dCTP	2´-Deoxycytidine-5´-triphosphate
dGTP	2´-Deoxyguanosine-5´-triphosphate
dTTP	2´-Deoxythymidine-5´-triphosphate
dUTP	2´-Deoxyuridine-5´-triphosphate
GTP	Guanosine-5´-triphosphate
IDP	Inosine-5'-diphosphate
ITP	Inosine-5´-triphosphate
mCAP	P1-(5´-7-methyl-guanosyl) P3-(5´-(guanosyl))triphosphate
N-2-Me-dGTP	N2-methyl-2'-deoxyguanosine -5'-triphosphate
NAD+	β-Nicotinamide adenine dinucleotide
NADPH	β-Nicotinamide adenine dinucleotide phosphate – reduced
